# Not out of the woods yet: “Diabetic neuropathy” or “neuropathy associated with diabetes”?

**DOI:** 10.1111/jdi.13780

**Published:** 2022-03-14

**Authors:** Tatsuhito Himeno, Hideki Kamiya, Jiro Nakamura

**Affiliations:** ^1^ Department of Innovative Diabetes Therapy Aichi Medical University School of Medicine Nagakute Japan; ^2^ Division of Diabetes Department of Internal Medicine Aichi Medical University School of Medicine Nagakute Japan

## Abstract

The most frequent diabetic complication, diabetic neuropathy, lacks accessible objective assessments. The concept and definition of diabetic neuropathy should be rethought to achieve the successful development of diagnostic and therapeutic methods.

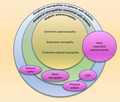

Distal symmetric polyneuropathy is the most prevalent diabetic complication. The current editorial refers to this distal symmetric polyneuropathy as diabetic polyneuropathy (DPN). The biggest problem that diabetologists must be aware of is that the diagnosis and treatment of DPN are far from satisfactory for both patients and healthcare professionals. On PubMed, over the past 50 years, the number of clinical trials for nephropathy has increased, which has established accessible objective screening tools, serum creatinine and urinary albumin (Figure 1). Thereafter, the number of studies on retinopathy, in which various treatments for angiogenesis have been proposed, has dramatically increased. In contrast, the number of clinical trials on DPN has remained at approximately half of the other two complications. The reason for the lowest number of trials on the most frequent complications can be attributed to the lack of accessible objective assessments and stagnant development of new therapies.

**Figure 1:**
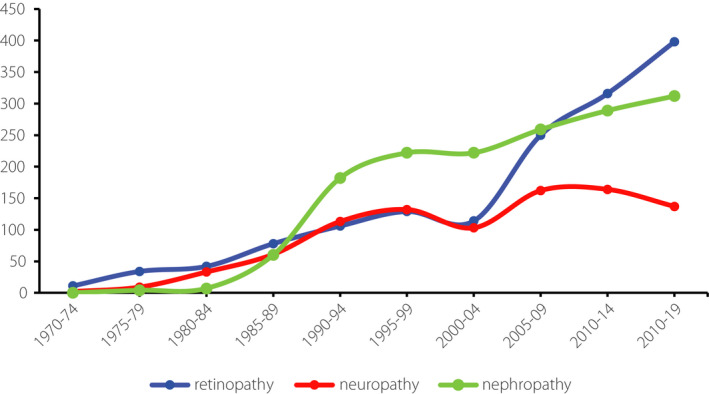
The number of clinical trials for diabetic complications. The *y*‐axis represents the number of clinical trials per 5 years. The *x*‐axis represents the period of observation.

There is no technical problem in establishing accessible objective assessments, but rather, we are in the final stage of determining the optimum solution. Composite indices of subjective symptoms, Achilles tendon reflex and vibration sensation using a tuning fork have been the major method of clinical diagnosis for DPN so far. However, it is difficult to detect neurodysfunction in the early stages of DPN using these low‐reproducibility indices.

To address this problem in diagnosis, many testing devices or methods for evaluating peripheral nerve dysfunction or morphological abnormality have been proposed. Although the nerve conduction study (NCS) is classically the most reliable functional test, the lack of standardized interpretation of its results has been a barrier to carrying out multicenter clinical trials. In Japan, standardization has been attempted in interpreting the results of NCS, and as a result, it is widely used in medical institutions as Baba’s classification on the severity of DPN.^1^ Although this classification would be of great benefit to define the outcomes of clinical trials for DPN, it has not cleared the barrier of availability.

However, the point‐of‐care NCS device, NC‐stat DPNCheck, which is designed to facilitate standardized electrode placement, is expected as a testing device that can eliminate this barrier. It has been verified in the past two decades that this device can partly replace the roles of conventional NCS. The high reproducibility in technical performance of this point‐of‐care device is superior to that of conventional NCS in which minor technical variations can yield inconsistent results. This device will provide accessible, objective and repetitive assessment of DPN, even in developing areas with limited medical resources. Various semiquantitative subjective tests other than this device have been proposed: Neuropad, NeuroQuick, Ipswich Touch Test, Vibratip and NerveCheck. These tests might also be used for diagnosis, along with DPNCheck. Additionally, among the tests for diabetic autonomic neuropathy, RR interval variability on the electrocardiogram is commonly available data. As the values of the variability have low reproducibility, it might be appropriate to use these data as an adjunct to the diagnosis of DPN, such as the use of urinary albumin in the diagnosis of diabetic nephropathy. To develop treatments for DPN, diagnostic criteria that are quantitative and objective, and can be easily applied in daily clinical practice should be established.

Insufficient understanding of the mechanism is the most important barrier to developing new therapies for DPN. As various mechanisms have been proposed, most researchers report that the pathogenesis of DPN consists of multiple factors, and intervention on a single factor is not sufficient to prevent the development and progression of DPN. Based on this pathogenetic theory, the pathogenetic factors of DPN can be roughly divided into two: peripheral ischemia and metabolic changes associated with hyperglycemia. However, no individual pathogenetic hypothesis has been proven to be superior to other hypotheses. The lack of sufficient evidence for each hypothesis might be caused by insufficient therapeutic effectiveness of each intervention.

We, researchers, should responsibly verify our unique hypothesis without being affected by research trends in academia. For example, researchers should not blindly flock to large currents, such as oxidative stress; rather, to understand the complex factors that regulate oxidative stress, we should carefully explore the cascade of unique molecules. Apart from the detailed studies that explore the pathology, we should also proceed with studies that compare the effectiveness of various suggested therapies. First, the impact of recent advances in glycemic control on the development and progression of DPN should be investigated. Thereafter, the additive effects of the existing approved drugs, α‐lipoic acid and aldose reductase inhibitor, should be evaluated.

When carrying out a clinical trial, we should keep in mind using accessible objective assessments and to have a long‐term observation period. In these tests, as the symptoms and signs, which are often included as a primary end‐point due to the request by an examination authority in each country, impair objectivity and do not necessarily indicate an exacerbation of DPN stages, these symptoms and signs should not be used as primary end‐points. Accumulating experience through these trials of hypoglycemic therapies and the approved drugs will enable us to develop stronger strategies when planning clinical trials for new drugs.

We now need to rethink the concept and definition of DPN to avoid future confusion in anticipation of the successful development of diagnostic and therapeutic methods for DPN. Although the most common clinical picture of diabetic polyneuropathy is distal symmetry, the current comprehensive classifications of diabetic neuropathy proposed by each country or organization include focal neuropathies. However, it is not guaranteed that these neuropathies with diverse clinical features have a common pathology. Rather, to promote accurate scientific verification, it would be better to consider that each individual neuropathy has a unique pathological mechanism. Therefore, is it appropriate to define diabetic neuropathy as a term that refers to DPN and tentatively call other neuropathies “neuropathy associated with diabetes” (Figure 2)? A comprehensive classification scheme for the diabetic neuropathies in the current position statement by the American Diabetes Association^2^ includes neuropathies that might not be directly caused by diabetes. Therefore, the term “neuropathy associated with diabetes” would be more accurate. By changing the disease name to neuropathy with diabetes, it becomes clearer that the pathophysiology of the diseases included here is diverse, and it is expected that a unique approach tailored to each neuropathy will be developed.

**Figure 2:**
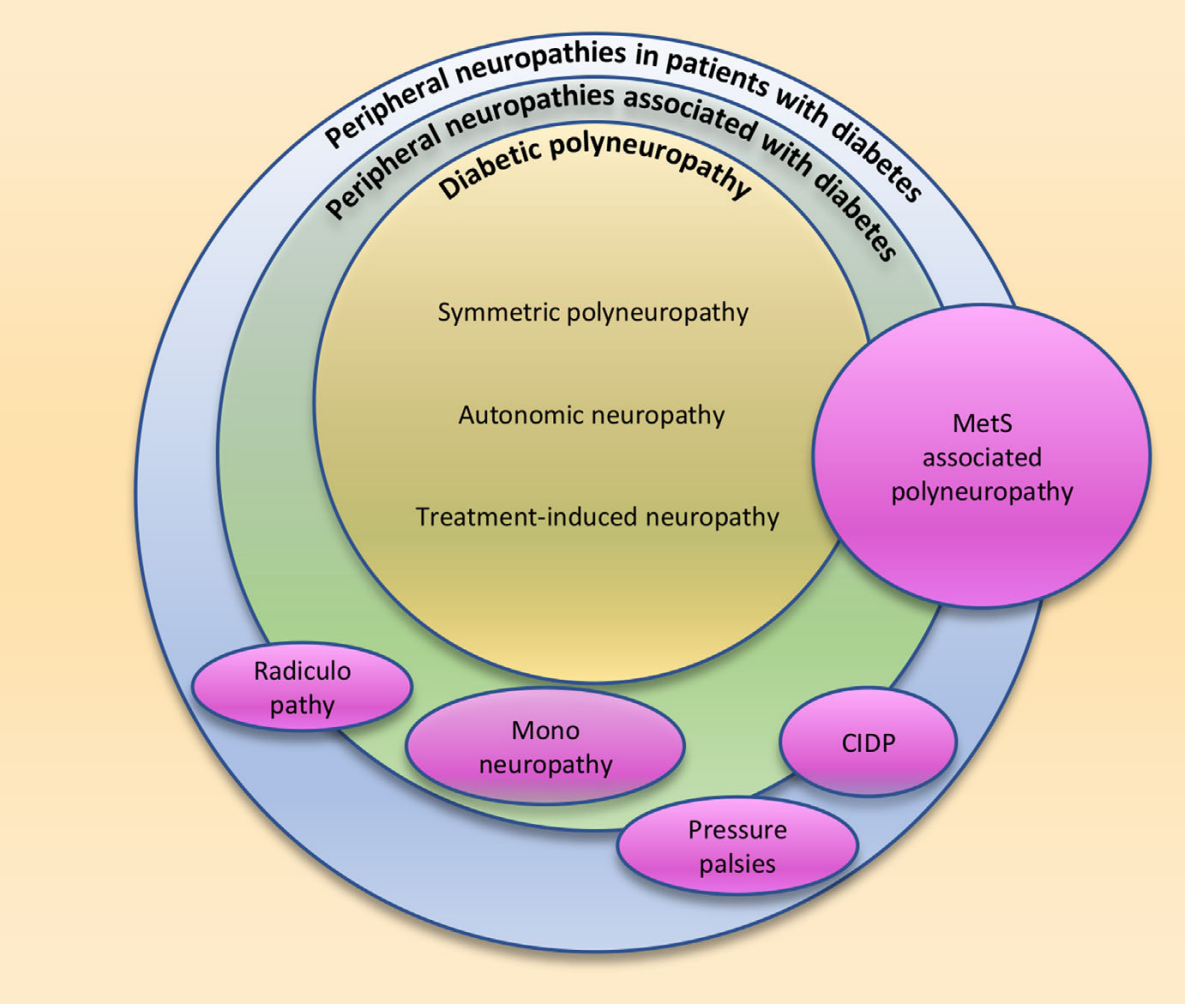
The classification of peripheral neuropathies in patients with diabetes. The schematic Venn diagram of the classification. Each circle represents an assumption of the similar etiology. CIDP, chronic inflammatory demyelinating polyneuropathy; MetS, metabolic syndrome.

We scientists should not forget that the way of thinking among humans, including patients, is irrational. People do not feel the necessity to immediately address potentially progressive illnesses, such as diabetes and DPN, even if they understand that these illnesses greatly impair their career choices in life. As a result, many government agencies, researchers, clinicians and patients pretend not to see the diseases. Although necessity is the mother of invention, we have a responsibility to accurately diagnose and treat DPN before the patient becomes aware of it. We must now take action to improve diagnosis and treatment of DPN.
